# Pretreatment clinical and hematological predictors of efficacy and immune-related adverse events in patients with advanced non-small cell lung cancer receiving first-line chemotherapy combined with immune checkpoint inhibitors

**DOI:** 10.1186/s12885-026-15733-9

**Published:** 2026-02-12

**Authors:** Shun Matsuura, Kensuke Kita, Kyohei Matsushita, Takumi Nagasaki, Ryo Suzuki, Yuya Yamamoto, Kotaro Yamada, Ryuuichi Nakamura, Norimichi Akiyama, Kazuki Tanaka, Naoki Koshimizu

**Affiliations:** Division of Respiratory Internal Medicine, Fujieda Municipal General Hospital, 4-1-11, Surugadai, Fujieda, Shizuoka 426-8677 Japan

**Keywords:** Advanced non-small cell lung carcinoma, ECOG-PS, Number of metastases, Immune-related adverse events

## Abstract

**Background:**

The combination of immune checkpoint inhibitors (ICI) and platinum-based chemotherapy has rapidly become the standard first-line treatment for advanced or metastatic non-small cell lung cancer (NSCLC). However, identifying reliable predictive factors of treatment response remains a significant clinical challenge. This study comprehensively analyzed pretreatment predictive factors in patients with advanced lung cancer who received first-line ICI–chemotherapy combination therapy.

**Methods:**

We retrospectively analyzed clinical data from 100 patients with advanced NSCLC who received first-line ICI–chemotherapy. Univariate and multivariate Cox proportional hazards regression analyses were used to identify prognostic factors for progression-free survival (PFS) and overall survival (OS). Univariate logistic regression analysis was used to identify factors affecting the incidence of immune-related adverse events (irAEs).

**Results:**

Multivariate analysis for PFS identified Eastern Cooperative Oncology Group performance status (ECOG-PS), number of metastases, lactate dehydrogenase level, cytokeratin 19 fragment antigen level, and programmed death-ligand 1 expression as independent predictors of PFS. Multivariate analysis for OS identified ECOG-PS, number of metastases, and neutrophil-to-lymphocyte ratio as significant independent predictors of OS. Patients with liver metastases showed a significantly lower incidence of irAEs than patients without liver metastases (8.3% vs. 29.7%, *P* = 0.013). The occurrence of irAEs was associated with longer PFS and OS.

**Conclusion:**

This study identified pretreatment clinical and hematological factors that could predict the efficacy of ICI–chemotherapy. Furthermore, the absence of liver metastases was associated with a higher incidence of irAEs. These results underscore the critical need for individualized treatments and potential adjustment of strategies for high-risk groups to achieve a long-term durable response and better survival.

**Supplementary Information:**

The online version contains supplementary material available at 10.1186/s12885-026-15733-9.

## Background

The combination of immune checkpoint inhibitors (ICI) and platinum-based chemotherapy has become the standard first-line treatment for patients with advanced or metastatic non-small cell lung cancer (NSCLC) without actionable driver mutations [1–3]. However, despite their combined efficacy, identifying reliable predictive factors for treatment response remains a significant clinical challenge. Notably, a subset of patients treated with ICIs or ICI–chemotherapy combinations achieve remarkably long-term responses and durable survival. To identify patients most likely to experience long-term treatment benefits, pretreatment predictive factors must be comprehensively understood.

Extensive studies have attempted to identify potential markers of ICI efficacy, evaluating clinical findings, blood-based parameters, and tumor-based immunohistochemistry, such as programmed death ligand 1 (PD-L1) expression and tumor mutation burden [4–6]. Furthermore, the development of immune-related adverse events (irAEs) may be correlated with improved overall survival (OS) in advanced NSCLC [7]. Nevertheless, existing reports have not consistently established pretreatment demographic, peripheral laboratory, or tumor characteristics as reliable predictors for the development of irAEs [8–10]. Combining chemotherapy with ICI further complicates the clinical situation, as this approach must consider not only the anti-tumor effect but also the possibility of irAEs. Pretreatment predictive factors related to combination therapy may be useful to oncological clinicians.

Studies specifically addressing predictors for chemotherapy and ICI combination therapy remains limited due to the wide range of additional elements introduced by such treatment combinations. Therefore, our study analyzed the clinical data of patients with advanced lung cancer who received first-line ICI–chemotherapy combination therapy. The primary objective was to identify pretreatment factors that can predict both progression-free survival (PFS) and OS, with specific emphasis on identifying markers associated with long-term durable response. Furthermore, we investigated pretreatment predictive factors associated with the development of irAEs.

## Patients and methods

### Study population

We retrospectively analyzed data from 100 patients with advanced NSCLC admitted to the Fujieda Municipal General Hospital from February 2018 to September 2024 on September 17, 2025. The inclusion criteria were as follows: (a) age ≥ 18 years old; (b) advanced (stage IIIC, IVA, or IVB) disease; (c) available pretreatment laboratory data; (d) complete clinicopathological information; (e) Eastern Cooperative Oncology Group performance status (ECOG-PS) of 0–1; and (f) patients who received a combination of chemotherapy and ICI as a first-line treatment for NSCLC. Patients who had undergone first-line treatment with a targeted therapy or had a known hematologic disorder were excluded. Since this retrospective study only involved de-identified patient data, with no potential harm or impact on patient care, informed consent was waived. The study was approved by the medical ethics committee of the Fujieda Municipal General Hospital (R07-29) and was conducted in accordance with the Declaration of Helsinki of the World Medical Association.

### Clinical data collection

Baseline characteristics were collected using an electronic medical records system, including sex, age, smoking status, ECOG-PS, pathological diagnosis, clinical stage, number of metastatic sites, and therapeutic information. The ECOG-PS scores were defined as follows: 0 indicated high activity and 1 indicated restriction in strenuous activity. Cancer staging was based on the eighth edition of the Tumor, Node, Metastasis Classification of the International Union Against Cancer [11]. The number of metastases was defined as the total count of individual metastatic lesions identified on pretreatment imaging. To minimize the impact of outliers among patients with extensive systemic disease, the maximum number was capped at 10 lesions for the analysis.

### Specimen collection and tumor marker assays

Serum carcinoembryogenic antigen (CEA) concentrations were quantitatively determined using a CEA assay kit (Abbott Japan, Tokyo, Japan). Cytokeratin 19 fragment antigen (CYFRA21-1) was detected using a commercially available electrochemiluminescence immunoassay kit (Roche Diagnostics Corp, Tokyo, Japan) according to the manufacturer’s instructions. Lactate dehydrogenase (LDH) levels and C-reactive protein (CRP) levels were measured using standard assays of pretreatment blood samples. The following equation was used to calculate the geriatric nutritional risk index (GNRI): 14.89 × serum albumin (g/dL) + 41.7 × (body weight/ideal body weight). Ideal body weight was measured using height and body mass index (22.0 kg/m2). Body weight/ideal body weight was set at 1 when the patient’s body weight exceeded their ideal body weight, as described previously [12]. Prognostic nutritional index (PNI) was calculated using the following formula: 10 × serum albumin (g/dL) + 0.005 × peripheral lymphocyte count (cells/mm3) in peripheral blood [13]. The neutrophil-to-lymphocyte ratio (NLR) was defined as the absolute number of neutrophils divided by the absolute number of lymphocytes. The following adverse events were defined as irAEs: thyroiditis, pneumonitis, dermatitis, hepatitis, colitis, nephritis, pituitary inflammation, and hemophagocytosis.

### PD-L1 immunohistochemical staining and evaluation of PD-L1 expression

PD-L1 expression in tumors was assessed via immunohistochemistry (IHC) using the 22C3 pharmDx assay on pretreatment tumor samples at a commercial clinical laboratory (SRL Inc., Tokyo, Japan). PD-L1 expression was defined as the percentage of at least 100 viable tumor cells exhibiting complete or partial membranous staining. PD-L1 expression was analyzed by pathologists at the commercial laboratory in accordance with the assay criteria.

### Statistical analysis

Data are expressed as median (interquartile range). Differences between the two or three groups were tested using the Fisher’s exact test for discrete variables and the Mann–Whitney U-test or Kruskal–Wallis one-way analysis for continuous variables. As an exploratory analysis, receiver operating characteristic (ROC) curves were used to estimate the tentative cutoff value of hematological markers and each nutritional index for predicting 1-year PFS and 2-year OS. PFS was defined as the time from the first infusion of the first-line chemotherapy protocol to the date of disease progression or death, whichever occurred first. Disease progression was assessed according to the Response Evaluation Criteria in Solid Tumors, version 1.1. Radiographic assessments, including computed tomography or magnetic resonance imaging, were performed every 8–12 weeks or more frequently if clinically indicated. The treating physicians determined progression based on a review of the radiographic findings. OS was defined as the time from treatment to the date of death from any cause. For each level of disease severity, PFS and OS were analyzed using the log-rank test for Kaplan–Meier survival analysis. To identify independent prognostic factors, we employed univariate and multivariate Cox proportional hazards regression models, estimating hazard ratios (HRs) and 95% confidence intervals (CIs). The variables analyzed included age, sex, ECOG-PS, smoking history, pathology, PD-L1 expression, clinical stage, number of metastases, first-line therapy, CRP, LDH, CEA, CYFRA, NLR, GNRI, and PNI. After confirming the proportional hazards assumption, variables with a p-value < 0.05 in the univariate analysis were included in the multivariate Cox regression model to identify independent predictors of PFS and OS. All statistical tests were two-way and were performed using EZR (Saitama Medical Center, Jichi Medical University) [14], a graphical user interface for R (R Foundation for Statistical Computing, ver. 4.3.1).

## Result

### Patients’ characteristics

This study analyzed data from 100 patients with advanced lung cancer who received first-line chemotherapy plus ICI (Table 1). The median age was 71 years (range: 48–82 years). The majority of the patients was male (81%) and had a smoking history (91%). 61% of patients had an ECOG-PS of 0, while 39% had an ECOG-PS of 1. More than half of the patients (54%) had stage ⅣB disease. The distribution of PD-L1 expression was as follows: 41.5% with < 1%, 26.6% with 1%–49%, and 31.9% with > 50%. The most common treatment regimen administered to 68% of patients was pembrolizumab plus pemetrexed–platinum chemotherapy. Regarding the post-progression status of all 100 patients, 21 (21%) were still receiving first-line treatment at the data cutoff point, 43 (43%) had proceeded to second-line therapy, and 36 (36%) were receiving best supportive care.

**Table 1:** Clinical characteristics of the patients

Factor	All cases
	*n* = 100
Gender	
Male / Female	81 / 19
Age	
Median (range), years	71 (48–82)
Smoking status	
Never / ever or current	9 / 91
ECOG-PS	
0 / 1	61 / 39
Pathology	
Adenocarcinoma / Squamous / others	66 / 25 / 9
PD-L1 expression	
1%< / 1%–49% / ≤50% / unknown	39 / 25 / 30 / 6
Stage	
Ⅲ, ⅣA / ⅣB	46 / 54
The number of metastases	
Median (range)	2 (0–10 or more)
Brain metastases	
Yes / No	23 / 77
Liver metastases	
Yes / No	22 / 78
CRP	
Median (range)	2.0 (0.03–36.3)
LDH	
Median (range)	222 (124–1855)
CEA	
Median (range)	7.3 (0.9–3314)
CYFRA	
Median (range)	5.3 (1.0–75.0)
NLR	
Median (range)	4.5 (1.6–31.2)
GNRI	
Median (range)	91.6 (52.0-117.1)
PNI	
Median (range)	40.9 (22.6–57.0)
Treatment	
Platinum + PEM + Pembro / Platinum + nab-PTX or PTX + Pembro / Platinum + nab-PTX+ Atezo	68 / 27 / 5

*ECOG-PS* Eastern Cooperative Oncology Group performance status, *PD-L1* Programmed cell death 1 - ligand 1, *NLR* Neutrophil-to-lymphocyte ratio, *GNRI* Geriatric nutritional risk index, *PNI* Prognostic nutritional index

### Predictive factors for progression-free survival

**Fig. 1:**
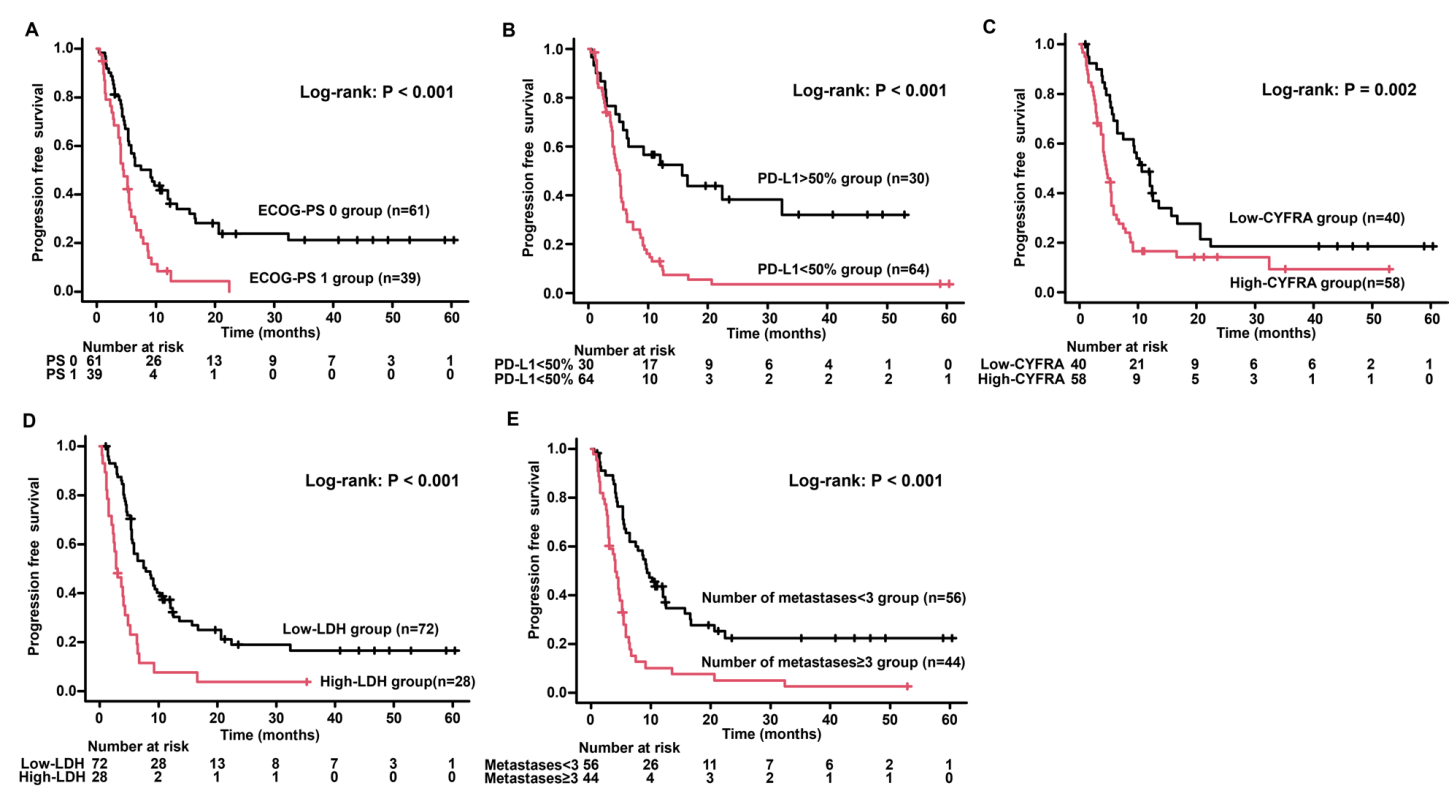
Kaplan–Meier curve analysis of PFS for patients with advanced non–small cell lung cancer treated with first-line immune-chemotherapy. PFS based on (**A**) ECOG-PS, (**B**) PD-L1 expression, (**C**) CYFRA, (**D**) LDH, and (**E**) number of metastases. PFS, progression-free survival; ECOG-PS, Eastern Cooperative Oncology Group performance status; PD-L1, programmed death ligand 1; CYFRA, cytokeratin 19 fragment antigen; LDH, lactate dehydrogenase

The median follow-up time was 11.9 months. The cutoff values for various factors were determined using ROC curves for PFS (Supplementary Figure S1). The cutoffs for LDH, CRP, CEA, CYFRA, NLR, GNRI, and PNI were 282, 0.65, 13.2, 3.5, 7.26, 87.8, and 40.2, respectively. Univariate analysis identified several factors significantly associated with a shorter PFS (Table 2), which were a high ECOG-PS score, low PD-L1 expression, stage IVB disease, a higher number of metastases, the presence of liver metastases, low GNRI, and high levels of LDH, CEA, CYFRA, and NLR. On multivariate analysis, the significant independent predictors of a shorter PFS were ECOG-PS of 1 (HR = 2.307; 95% CI, 1.388–3.836; *P* = 0.001), a high number of metastases (HR = 4.388; 95% CI, 1.601–12.03; *P* = 0.004), high LDH levels (HR = 2.929; 95% CI, 1.664–5.157; *P* < 0.001), and high CYFRA levels (HR = 1.913; 95% CI, 1.051–3.484; *P* = 0.033). Patients with PD-L1 expression score > 50% had a significantly lower risk of disease progression than other PD-L1 expression scores (HR = 0.261; 95% CI, 0.134–0.510; *P* < 0.001). PFS curves stratified by various baseline factors are shown in Fig. 1. Patients with a favorable ECOG-PS demonstrated a significantly longer median PFS (9.1 months vs. 4.5 months) compared with those with ECOG-PS 1. The estimated 1-year PFS rates were 41.8% for the ECOG-PS 0 group versus 8.4% for the ECOG-PS 1 group, a significant disparity in long-term efficacy. The PD-L1 expression > 50% group achieved a median PFS of 15.7 months and a 1-year PFS rate of 56.7%. In contrast, the low PD-L1 expression group had a median PFS of 5.2 months and a 1-year PFS rate of 13.0%, thereby demonstrating the superiority of high PD-L1 expression. Furthermore, patients with low CYFRA levels had a more favorable median PFS (10.7 months vs. 4.5 months) and 1-year PFS rate (48.6% vs. 16.6%) than those with high CYFRA levels. Similarly, the median PFS for the low LDH group was 7.8 months, which is longer than the 2.9 months observed in the high LDH group. Furthermore, patients with fewer metastatic sites had a median PFS of 9.4 months, suggesting a statistically favorable prognosis compared with 4.0 months for those with numerous metastatic sites.

**Table 2:** Prognostic factors for progression-free survival identified by univariate and multivariate Cox proportional hazards analyses

		Univariate analysis			Multivariate analysis		
		HR	95%CI	*p*-value	HR	95% CI	*p*-value
Age	70< / ≤70	1.240	0.796–1.929	0.431			
Gender	Male / Female	0.857	0.495–1.483	0.582			
ECOG-PS	0 / 1	2.448	1.545–3.878	< 0.001	2.307	1.388–3.836	0.001
Smoking	never / ever	1.139	0.534–2.429	0.735			
Pathology	Non squamous / Squamous	1.322	0.794–2.202	0.282			
PD-L1 expression	0%-49% / ≤50%	0.340	0.197–0.588	< 0.001	0.261	0.134–0.510	< 0.001
Stage	ⅢC・ⅣA / ⅣB	1.903	1.217–2.976	0.004	0.575	0.214–1.547	0.273
The number of metastases	3< / ≤3	2.819	1.795–4.427	< 0.001	4.388	1.601–12.03	0.004
Brain metastases	No / Yes	1.428	0.860–2.368	0.167			
Liver metastases	No / Yes	1.989	1.191–3.320	0.008	0.615	0.295–1.281	0.194
LDH	282< / ≤282	2.910	1.804–4.694	< 0.001	2.929	1.664–5.157	< 0.001
CRP	0.65< / ≤0.65	1.527	0.932–2.499	0.095			
CEA	13.2< / ≤13.2	2.314	1.459–3.670	< 0.001	1.536	0.917–2.571	0.102
CYFRA	3.5< / ≤3.5	2.035	1.281–3.232	0.002	1.913	1.051–3.484	0.033
NLR	7.26< / ≤7.26	1.970	1.148–3.383	0.013	1.602	0.882–2.907	0.121
GNRI	87.8< / ≤87.8	0.598	0.376–0.953	0.030	0.651	0.443–1.691	0.139
PNI	40.2< / ≤40.2	0.675	0.435–1.047	0.079			

*PFS* Progressive free survival, *ECOG-PS* Eastern Cooperative Oncology Group performance status, *PD-L1* Programmed cell death 1 - ligand 1, *NLR* Neutrophil-to-lymphocyte ratio, *GNRI* Geriatric nutritional risk index, *PNI* Prognostic nutritional index

### Predictive factors for overall survival

**Fig. 2:**
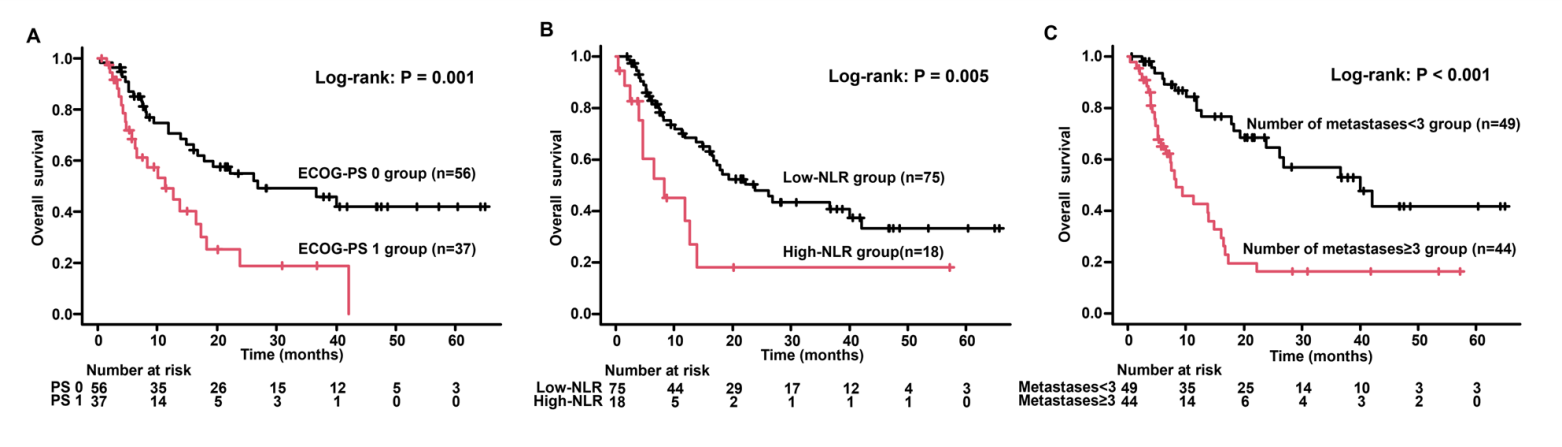
Kaplan–Meier curve analysis of OS for patients with advanced non–small cell lung cancer treated with first-line immune-chemotherapy. OS based on (**A**) ECOG-PS, (**B**) number of metastases, and (**C**) NLR. OS, overall survival; ECOG-PS, Eastern Cooperative Oncology Group performance status; NLR, neutrophil-to-lymphocyte ratio

Seven patients who had less than 2 years of follow-up from the start of treatment were excluded from the OS analysis, resulting in a final total of 93 patients. Cutoff values for various factors in relation to a 2-year OS were defined using ROC curves. The cutoff values for LDH, CRP, CEA, CYFRA, NLR, GNRI, and PNI were 295, 0.77, 16.2, 4.5, 7.26, 99.3, and 42.2, respectively (Supplementary Figure S2). Factors identified as significantly associated with a shorter OS on univariate analysis were ECOG-PS 1, presence of liver metastasis, more than three metastases, stage ⅣB disease, and elevated levels of CRP, CEA, CYFRA, LDH, and NLR (Table 3). Multivariate analysis identified ECOG-PS 1 (HR = 2.014; 95% CI, 1.102–3.682; *P* = 0.022), a high number of metastases (HR = 3.683; 95% CI, 1.048–12.94; *P* = 0.042), and high NLR levels (HR = 2.474; 95% CI, 1.079–5.67; *P* = 0.032) as significant independent predictors of shorter OS. Fig. 2 shows the OS curves based on ECOG-PS, number of metastases, and NLR. These prognostic factors also demonstrated significant associations with OS. Patients with ECOG-PS 0 had better survival, with a median OS of 26.8 months compared with 11.3 months for patients with ECOG-PS 1. The corresponding 2-year OS rates were higher for ECOG-PS 0 (54.8% vs. 18.9%). Patients with less than three metastatic sites had a median OS of 40.0 months and a 2-year OS rate of 64.5%, drastically exceeding those with three or more metastatic sites (median OS: 8.3 months; 2-year OS rate: 16.3%). The low NLR group achieved a median OS of 23.9 months (2-year OS rate: 48.0%), which was longer than the high NLR group’s median OS of 8.3 months (2-year OS rate: 18.0%). A detailed summary of the statistics and patient characteristics stratified by key prognostic biomarkers are provided in Supplementary Table S1.

**Table 3:** Prognostic factors for overall survival identified by univariate and multivariate Cox proportional hazards analyses

		Univariate analysis			Multivariate analysis		
		HR	95%CI	*p*-value	HR	95% CI	*p*-value
Age	70< / ≤70	1.653	0.9227–2.962	0.091			
Gender	Male / Female	0.942	0.468–1.895	0.864			
ECOG-PS	0 / 1	2.442	1.365–4.371	0.002	2.014	1.102–3.682	0.022
Smoking	never / ever	1.915	0.593-6,177	0.276			
Pathology	Non squamous / Squamous	0.975	0.476–1.991	0.944			
PD-L1 expression	0%-49% / ≤50%	1.802	0.925–3.508	0.083			
Stage	ⅢC・ⅣA / ⅣB	2.567	1.399–4.711	0.002	0.610	0.160–2.318	0.468
The number of metastases	3< / ≤3	3.380	1.871–6.104	< 0.001	3.683	1.048–12.94	0.042
Brain metastases	No / Yes	1.673	0.897–3.120	0.105			
Liver metastases	No / Yes	2.017	1.041–3.906	0.037	0.856	0.365–2.008	0.721
LDH	295< / ≤295	2.316	1.205–4.454	0.011	1.638	0.772–3.476	0.198
CRP	0.77< / ≤0.77	1.874	1.011–3.474	0.046	1.227	0.590–2.551	0.584
CEA	16.2< / ≤16.2	2.042	1.123–3.712	0.019	1.785	0.886–3.593	0.104
CYFRA	4.5< / ≤4.5	2.027	1.129–3.637	0.017	1.340	0.676–2.655	0.401
NLR	7.26< / ≤7.26	2.565	1.293–5.088	0.007	2.474	1.079–5.670	0.032
GNRI	99.3< / ≤99.3	0.591	0.311–1.123	0.108			
PNI	42.2< / ≤42.2	0.827	0.467–1.464	0.515			

*OS* Overall survival, *ECOG-PS* Eastern Cooperative Oncology Group performance status, *PD-L1* Programmed cell death 1 - ligand 1, *NLR* Neutrophil-to-lymphocyte ratio, *GNRI* Geriatric nutritional risk index, *PNI* Prognostic nutritional index

### Predictive factors for immune-related adverse events

**Fig. 3:**
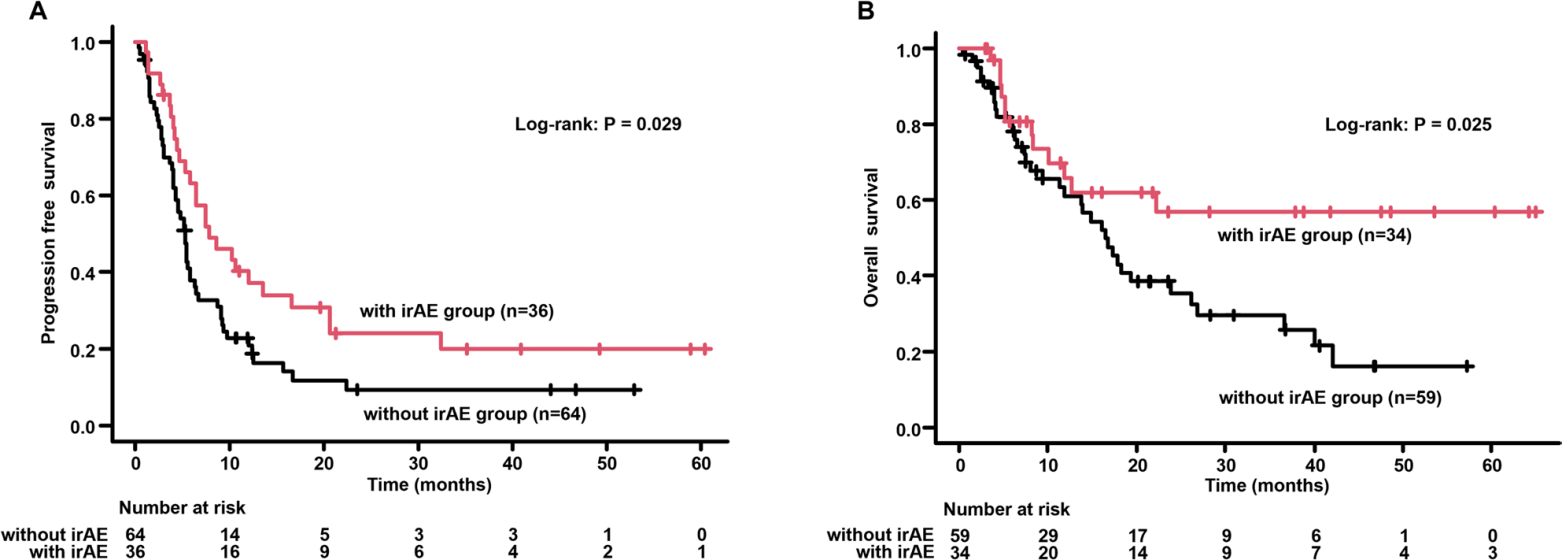
Kaplan–Meier curve analysis of PFS and OS for patients with advanced non–small cell lung cancer with or without irAEs. **A** PFS based on irAEs; (**B**) OS based on irAEs. PFS, progression-free survival; OS, overall survival; irAEs, immune-related adverse events

The frequency of irAEs is summarized in Table 4. The most common irAE was pneumonitis at 14%, followed by rashes (7%), endocrine disorders (6%), and hepatitis (5%). As shown in the univariate analysis in Fig. 3, the occurrence of irAEs is associated with prolonged PFS and OS, which is consistent with previously reported results. In an exploratory analysis of clinical factors associated with irAEs, a lower incidence of irAEs occurred in patients with than without liver metastases (8.3% vs. 29.7%, *P* = 0.013). No other clinical factors were significantly associated with the incidence of irAEs (Table 5).

**Table 4:** Incidence and profiles of immune-related adverse events

Event	Any grade *n* = 36		Severe irAE *n* = 16	
Pneumonitis	15	(41.6%)	4	(25.0%)
Dermatitis	8	(22.2%)	2	(12.5%)
Hepatitis	5	(13.8%)	1	(6.2%)
Colitis	4	(11.1%)	1	(6.2%)
Thyroiditis	4	(11.1%)	0	(0)
Nephritis	4	(11.1%)	2	(12.5%)
Adrenal insufficiency	2	(5.5%)	2	(12.5%)
Hemophagocytic	2	(5.5%)	2	(12.5%)
Other	2	(5.5%)	2	(12.5%)

**Table 5:** Clinical characteristics of patients with and without immune-related adverse events

Factor	Non-irAE	irAE	*P*-value
	*n* = 64	*n* = 36	
Gender			
Male / Female	54 / 10	27 / 9	0.293
Age			
Median (range), years	71 (48–82)	70 (53–82)	0.888
Smoking status			
Never / ever or current	4 / 62	5 / 31	0.277
ECOG-PS			
0 / 1	36 / 28	25 / 11	0.209
Pathology			
Adenocarcinoma / Squamous / others	41 / 17 / 6	25 / 8 / 3	0.897
PD-L1 expression			
1%< / 1%–49% / ≤50% / unknown	20 / 14 / 28 / 2	10 / 11 / 11 / 4	0.422
Stage			
Ⅲ, ⅣA / ⅣB	27 / 37	19 / 17	0.403
The number of metastases			
Median (range)	2 (0–10 or more)	1 (0–10 or more)	0.429
Brain metastases			
Yes / No	15 / 49	8 / 28	1.000
Liver metastases			
Yes / No	19 / 45	3 / 33	0.013
CRP			
Median (range)	2.5 (0.03–36.3)	1.4 (0.03–18.8)	0.270
LDH			
Median (range)	218 (124–1855)	225 (141–1513)	0.270
CEA			
Median (range)	6.9 (0.9–3314)	7.7 (1.2–983)	0.816
CYFRA			
Median (range)	5.4 (1.0–75.0)	4.8 (1.0–43.0)	0.412
NLR			
Median (range)	4.6 (1.6–31.2)	4.3 (1.6–11.9)	0.447
GNRI			
Median (range)	90.8 (64.2–117.0)	93.6 (52.0-112.0)	0.451
PNI			
Median (range)	40.9 (22.6–54.2)	40.9 (23.5–57.0)	0.527
Treatment			
Platinum + PEM + Pembro / Platinum + nab-PTX or PTX + Pembro / Platinum + nab-PTX+ Atezo	41 / 18 / 5	27 / 9 / 0	0.272

*ECOG-PS* Eastern Cooperative Oncology Group performance status, *PD-L1* Programmed cell death 1 - ligand 1, *NLR* Neutrophil-to-lymphocyte ratio, *GNRI* Geriatric nutritional risk index, *PNI* Prognostic nutritional index

## Discussion

This study serves as a real-world validation of several significant predictors of the effectiveness of ICI–chemotherapy combination therapy in patients with advanced NSCLC. Specifically, the identified pretreatment factors could independently predict PFS in these patients. While numerous investigations have examined ECOG-PS and PD-L1 expression, our findings serve as practical evidence supporting their utility in daily clinical practice, particularly within a Japanese cohort. In the present study, the number of metastatic lesions was an independent factor in PFS, indicating that not only tumor volume but also multiple organ metastases influence patient outcomes. CYFRA, a tumor marker reflecting tumor burden, was also an independent predictive factor for PFS. Previous studies have shown that serum CYFRA 21 − 1 levels are correlated with tumor size, lymph node metastasis, and disease stage [15]. Kataoka et al. [16] demonstrated that lower serum CYFRA 21 − 1 levels were associated with better PFS in patients with NSCLC receiving chemotherapy and immunotherapy combination therapy. Furthermore, elevated pretreatment LDH levels in NSCLC are associated with shorter OS and increased metastasis rates, especially in patients receiving ICIs [17, 18]. Our findings on the relevance of elevated CYFRA and LDH levels are consistent with these previous findings.

Multivariate analysis identified ECOG-PS 1, a high number of metastases, and high NLR levels as independent predictors of shorter OS. A retrospective study of 466 patients with NSCLC treated with ICIs showed that NLR > 3 was associated with poor OS and PFS in ICI-treated patients but not in chemotherapy-treated patients [19]. Previous studies have suggested that NLR expression is a strong indicator of survival in patients with NSCLC receiving ICI monotherapy [20]. Liao et al. [21] reported that high pretreatment NLR levels are an independent prognostic factor for poor PFS in ICI–chemotherapy combination therapy. The present study identified low NLR as an independent risk factor for OS but not for PFS. The analysis revealed that ECOG-PS and the number of metastases were important independent factors in predicting PFS and OS in patients treated with ICI–chemotherapy. Furthermore, ECOG-PS was closely associated with patient background factors such as age and GNRI (Supplementary Table S1). These factors may be intricately involved with ECOG-PS. A poor prognosis is associated with a higher ECOG-PS score, which was identified as an independent prognostic factor for PFS and OS. In particular, malnutrition due to low GNRI is closely correlated with ECOG-PS in patients with advanced NSCLC [22, 23]. Furthermore, it is important to situate our findings within the context of established prognostic scores used in the immuno-oncology era, such as the EPSILoN score and the Lung Immune Prognostic Index [24–26]. These scores integrate factors such as ECOG-PS, LDH, and the NLR to stratify risk among patients treated with ICIs. Although our results reinforce the prognostic relevance of these established components, they also provide real-world validation in a Japanese cohort receiving combination ICI–chemotherapy.

In our study, the group with more than three metastases was used as a surrogate for aggressive disease biology. This was supported by a higher frequency of metastases in organs with poor prognosis, such as the brain and liver, as well as significantly elevated markers of tumor burden and metabolic activity, including LDH, CEA, and CYFRA (Supplementary Table S1). Furthermore, a higher number of metastases was associated with disease progression and malignancy, including metastatic sites associated with poor prognosis and high tumor burden (high LDH/CEA/CYFRA levels). Although clinical stage was associated with survival in the univariate analysis, only the number of metastases remained an independent prognostic factor in the multivariate model. This indicated that metastatic burden more directly reflects the biological aggressiveness and treatment resistance of NSCLC than conventional stage grouping (IIIC-IVB) in patients receiving ICI plus chemotherapy. High tumor burden (> 3 organs involved at baseline) is also a prognostic factor for worse PFS in patients treated with ICI monotherapy [27]. Therefore, in advanced lung cancer, predicting the long-term response and survival rates is crucial for combination treatments of ICIs and chemotherapy. For high-risk groups, which are defined by a combination of multiple patient factors, such as ECOG-PS and the number of metastases, reflecting tumor metabolism, personalized medicine must be considered, including adjusting treatment strategies and intensity.

Our study revealed that in patients receiving ICI–chemotherapy, the group with liver metastases had a significantly lower incidence of irAEs than the group without it (13.6% vs. 42.3%). A large-cohort study previously identified factors associated with the development of irAEs, including age > 60 years, high PD-L1 expression, and the absence of bone metastases [9]. While our findings suggest that specific metastatic sites, such as liver metastases, may be associated with distinct patterns in the development of systemic irAEs, this observation remains exploratory given the small subgroup size. Unlike the larger cohort studied by Cook et al. [9], which included patients receiving ICI monotherapy, other combination therapies, and those with genetic mutations, our study was strictly limited to patients who received ICI and chemotherapy as first-line treatment. This specific focus may have created a unique immune profile due to chemotherapy and the liver tumor burden. Nevertheless, the link between the occurrence of irAEs and improved prognosis remains debatable in light of potential immortal time bias, and the specific impact of liver metastases on the occurrence of irAEs is not established. Further research using time-dependent analyses in larger cohorts is needed to determine whether this association reflects a true biological effect of the liver metastatic microenvironment or merely a cohort-specific finding.

This study has some limitations. First, this was a retrospective study conducted at a single institution, with a relatively small sample size of 100 patients, which may limit the generalizability of our results and increase the risk of overfitting in multivariate analyses. Given the retrospective nature of the study, no formal sample size calculation was performed. Consequently, the findings, particularly those regarding rare irAEs, should be interpreted as exploratory. Furthermore, although our study identified several independent prognostic factors, their combined clinical utility was not evaluated using decision curve analysis to avoid the risk of overfitting considering the limited sample size. Based on these exploratory findings, a logical future direction would be the development of an integrated risk score or predictive model that incorporates clinical, hematological, and tumor-specific factors. Such a model could offer more personalized prognostic information for patients receiving ICI–chemotherapy. Our statistical approach for variable selection in the multivariate analysis might have excluded some potential confounders. While we focused on variables with univariate significance to maintain model parsimony, factors such as smoking history and comorbidities should be more rigorously evaluated in larger, multi-center studies. Second, elevated tumor markers may differ depending on the tissue type. Third, a decrease in NLR was an independent risk factor for OS, but not for PFS. The NLR cutoff value in the current study was higher than those in previous reports, which raises questions on the validity of the ROC curve. The cutoff values for hematological markers and each nutritional index were determined using ROC curves based on 1-year PFS and 2-year OS, which may have introduced data-driven bias. In this single-center retrospective study, we emphasized clinical utility and model parsimony. Thus, we acknowledge that future studies should employ more robust methods, such as analyzing variables as continuous covariates, in larger, multi-center cohorts. Fourth, thyroid dysfunction was less prevalent among irAEs than previously reported, whereas the incidence of pneumonitis was high at 14%. The latter is consistent with those reported previously by Japanese studies at > 10% [28, 29]. The association between irAEs and improved survival must be interpreted with caution due to immortal time bias. Because patients must survive long enough to develop irAEs, this association may cause an overestimation of survival in the group with irAEs. As we did not perform time-dependent covariate analysis, this finding should be regarded as a clinical correlation rather than a confirmed causal relationship.

## Conclusion

In conclusion, our study provides a real-world validation of several clinical and hematological parameters as prognostic factors for advanced NSCLC treated with first-line chemo-immunotherapy. While factors such as ECOG-PS, tumor burden (including metastatic lesions), and inflammatory markers (NLR, LDH, CYFRA) are well-established, their integrated assessment remains essential for predicting survival in a Japanese clinical cohort. Our exploratory observations on the predictive value of irAEs and their association with liver metastases further underscore the importance of personalized monitoring. These results emphasize the importance of a personalized therapeutic approach in patients who have both patient-related factors, such as poor ECOG-PS, and tumor-related factors, such as high metastatic burden. 

## Supplementary Information

Supplementary Figure S1. ROC curve analysis for PFS in patients with advanced non–small cell lung cancer. ROC curves for A) LDH, B) CRP, C) CEA, D) CYFRA, E) NLR, F) GNRI, and G) PNI. ROC, receiver operating characteristic; PFS; progression-free survival; LDH, lactate dehydrogenase; CRP, C-reactive protein; CEA, carcinoembryogenic antigen; CYFRA, cytokeratin 19 fragment antigen; NLR, neutrophil-to-lymphocyte ratio; GNRI, geriatric nutritional risk index; PNI, prognostic nutritional index.

Supplementary Figure S2. ROC curves analysis for OS in patients with advanced non–small cell lung cancer. ROC curves for A) LDH, B) CRP, C) CEA, D) CYFRA, E) NLR, F) GNRI, and G) PNI. ROC; Receiver operating characteristic, OS; overall survival, NLR; neutrophil-to-lymphocyte ratio, GNRI; geriatric nutritional risk index, PNI; prognostic nutritional index.

Supplementary Table S1. Summary statistics of clinical parameters and biomarkers across patient subgroups.

## Data Availability

Due to privacy concerns, the raw data will not be fully disclosed. Incomplete raw data and various algorithmic codes are available upon request to the corresponding author.
