# Supplementary figures and images for: Pretreatment clinical and hematological predictors of efficacy and immune-related adverse events in patients with advanced non-small cell lung cancer receiving first-line chemotherapy combined with immune checkpoint inhibitors
Source: BMC Cancer. 2026 Feb 12;26:388. doi: 10.1186/s12885-026-15733-9 (PMC13014870; doi:10.1186/s12885-026-15733-9)

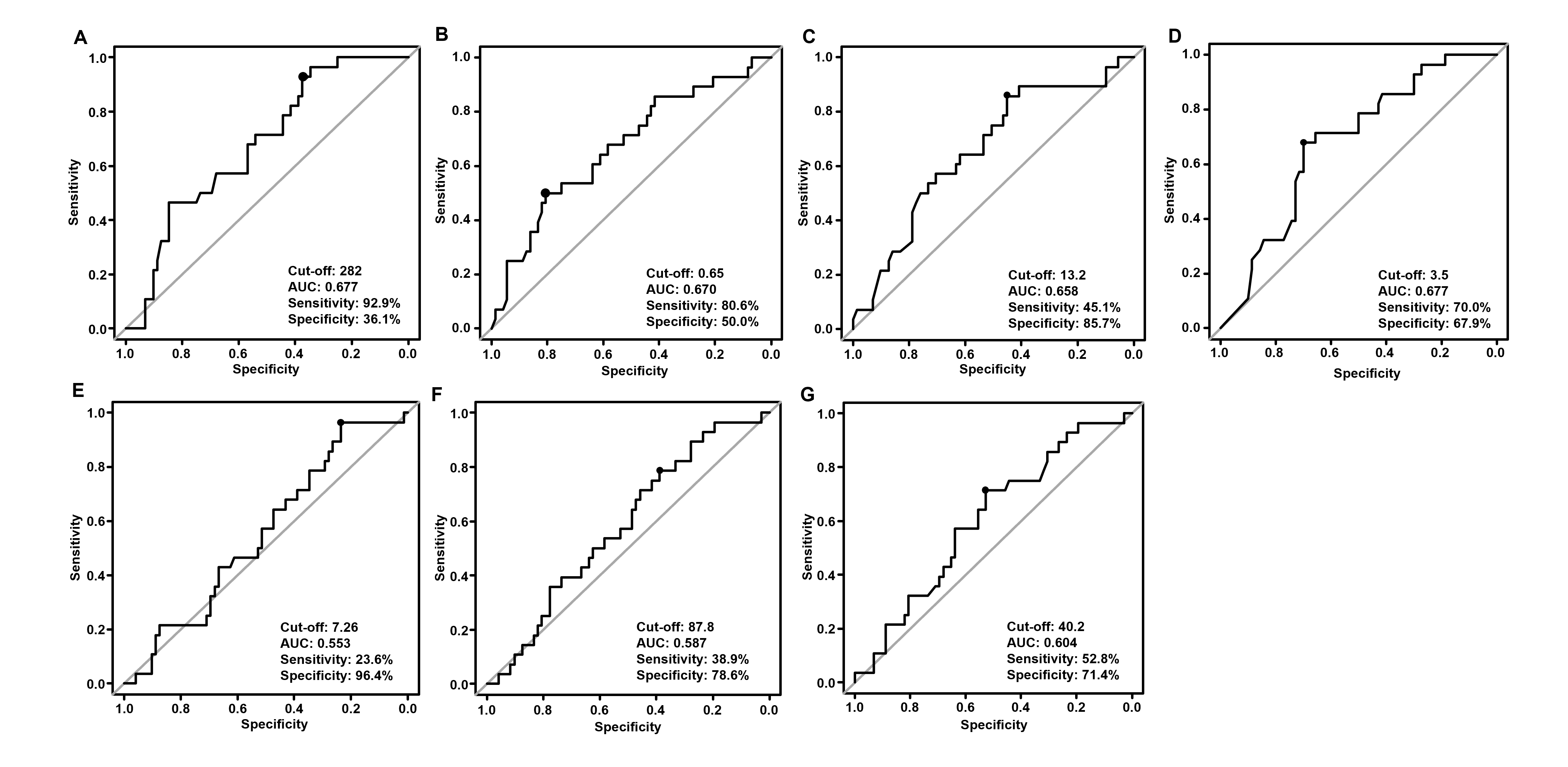

Supplement: Supplementary file 1 — Supplementary Figure S1. ROC curve analysis for PFS in patients with advanced non–small cell lung cancer. ROC curves for A) LDH, B) CRP, C) CEA, D) CYFRA, E) NLR, F) GNRI, and G) PNI. ROC, receiver operating characteristic; PFS; progression-free survival; LDH, lactate dehydrogenase; CRP, C-reactive protein; CEA, carcinoembryogenic antigen; CYFRA, cytokeratin 19 fragment antigen; NLR, neutrophil-to-lymphocyte ratio; GNRI, geriatric nutritional risk index; PNI, prognostic nutritional index. [file 12885_2026_15733_MOESM1_ESM.tif]

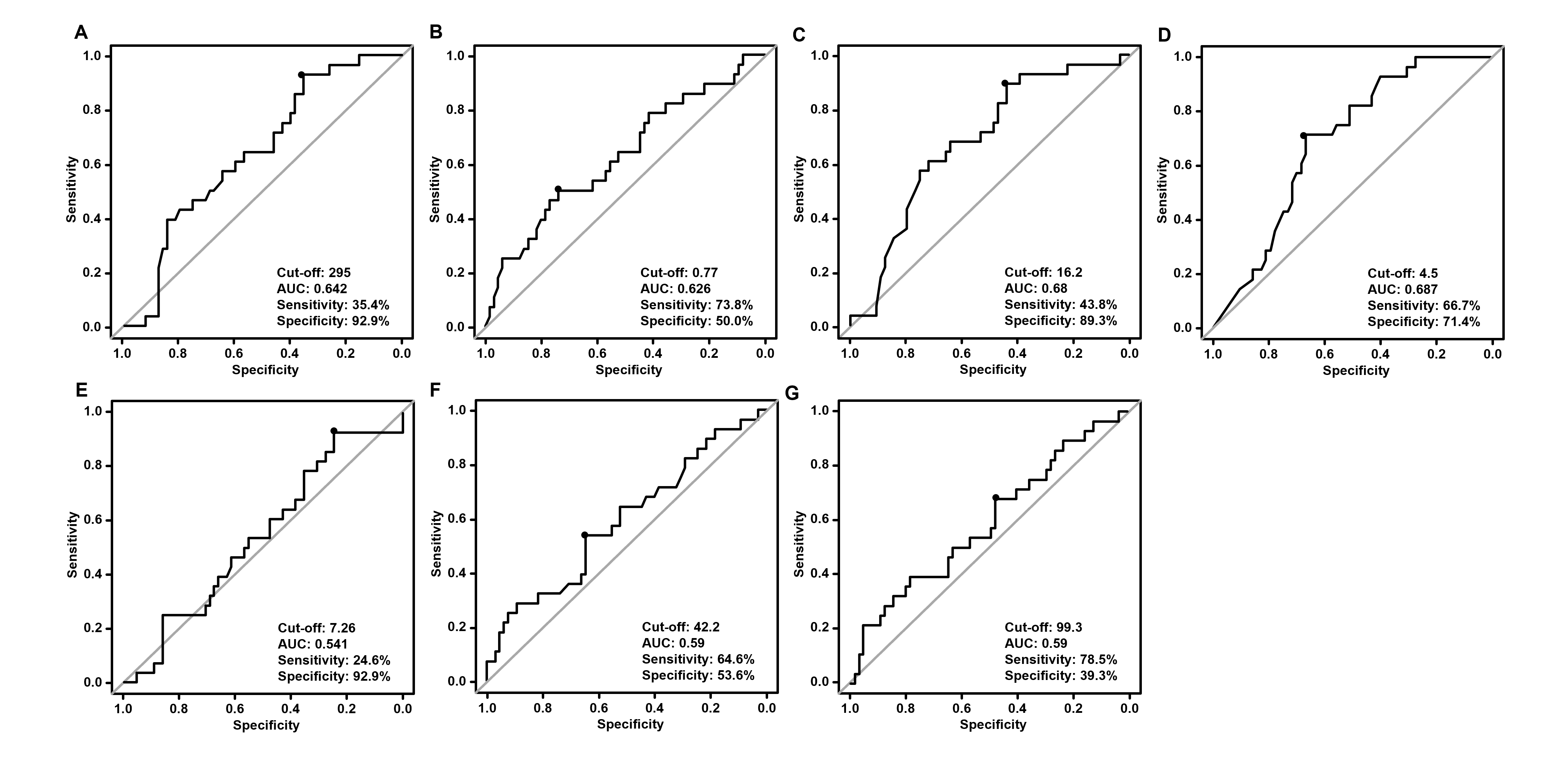

Supplement: Supplementary file 2 — Supplementary Figure S2. ROC curves analysis for OS in patients with advanced non–small cell lung cancer. ROC curves for A) LDH, B) CRP, C) CEA, D) CYFRA, E) NLR, F) GNRI, and G) PNI. ROC; Receiver operating characteristic, OS; overall survival, NLR; neutrophil-to-lymphocyte ratio, GNRI; geriatric nutritional risk index, PNI; prognostic nutritional index. [file 12885_2026_15733_MOESM2_ESM.tif]
