# Supplementary material for: Pretreatment clinical and hematological predictors of efficacy and immune-related adverse events in patients with advanced non-small cell lung cancer receiving first-line chemotherapy combined with immune checkpoint inhibitors
Source: BMC Cancer. 2026 Feb 12;26:388. doi: 10.1186/s12885-026-15733-9 (PMC13014870; doi:10.1186/s12885-026-15733-9)
Supplement: Supplementary file 3 — Supplementary Table S1. Summary statistics of clinical parameters and biomarkers across patient subgroups. [file 12885_2026_15733_MOESM3_ESM.docx]

**Supplementary Table S1.** **Summary statistics of clinical parameters and biomarkers across patient subgroups.**

| **Factor** | **ECOG-PS 0** | **ECOG-PS 1** | **P-value** |  | **PD-L1<50%** | **PD-L1≥50%** | **P-value** |
| --- | --- | --- | --- | --- | --- | --- | --- |
|  | **n = 61** | **n = 39** |  |  | **n = 64** | **n = 30** |  |
| **Gender** |  |  |  |  |  |  |  |
| **Male / Female** | **47 / 14** | **34 / 5** | **0.297** |  | **52 / 12** | **24 / 6** | **1.000** |
| **Age** |  |  |  |  |  |  |  |
| **Median (range), years** | **68 (50–81)** | **75 (48-82)** | **0.002** |  | **71 (53–81)** | **71 (50-80)** | **0.911** |
| **Smoking status** |  |  |  |  |  |  |  |
| **Never / ever or current** | **6 / 55** | **3 / 36** | **1.000** |  | **6 / 58** | **2 / 28** | **1.000** |
| **ECOG-PS** |  |  |  |  |  |  |  |
| **0 / 1** | **-** | **-** |  |  | **37 / 27** | **20 / 10** | **0.499** |
| **Pathology** |  |  |  |  |  |  |  |
| **Adenocarcinoma / Squamous / others** | **42 / 14 / 5** | **24 / 11/ 4** | **0.764** |  | **43 / 16 / 5** | **19 / 7 / 4** | **0.738** |
| **PD-L1 expression** |  |  |  |  |  |  |  |
| **1%< / 1%–49% / ≥50%** | **21 / 16 / 20** | **18 / 9 / 10** | **0.514** |  | **39 / 25 / 0** | **0 / 0 / 30** | **<0.001** |
| **Stage** |  |  |  |  |  |  |  |
| **Ⅲ, ⅣA / ⅣB** | **31 / 30** | **15 / 24** | **0.304** |  | **29 / 35** | **13 / 17** | **1.000** |
| **The number of metastases** |  |  |  |  |  |  |  |
| **Median (range)** | **1 (0-10 or more)** | **3 (0-10 or more)** | **0.436** |  | **2 (0-10 or more)** | **2 (0-10 or more)** | **0.756** |
| **Brain metastases** |  |  |  |  |  |  |  |
| **Yes / No** | **14 / 47** | **9 / 30** | **1.000** |  | **15 / 49** | **7 / 23** | **1.000** |
| **Liver metastases** |  |  |  |  |  |  |  |
| **Yes / No** | **13 / 48** | **9 / 30** | **1.000** |  | **15 / 49** | **7 / 23** | **1.000** |
| **CRP** |  |  |  |  |  |  |  |
| **Median (range)** | **1.6 (0.03-36.3)** | **2.7 (0.05-24.8)** | **0.220** |  | **2.1 (0.03-18.8)** | **2.8 (0.04-36.3)** | **0.443** |
| **LDH** |  |  |  |  |  |  |  |
| **Median (range)** | **206 (141-1855)** | **234 (124-1819)** | **0.044** |  | **220 (124-1855)** | **228 (141-453)** | **0.945** |
| **CEA** |  |  |  |  |  |  |  |
| **Median (range)** | **6.2 (0.9-3314)** | **8.3 (0.9-3314)** | **0.92** |  | **9.2 (0.9-3314)** | **4.2 (1.6-347)** | **0.095** |
| **CYFRA** |  |  |  |  |  |  |  |
| **Median (range)** | **4.4 (1.0-75.0)** | **6.2 (1.0-66.4)** | **0.266** |  | **4.7 (1.0-75.0)** | **6.3 (1.0-66.4)** | **0.309** |
| **GNRI** |  |  |  |  |  |  |  |
| **Median (range)** | **95.6 (64.2-117.1)** | **88.9 (52.0-113.7)** | **0.049** |  | **92.3 (52.0-117.0)** | **90.2 (65.6-108.4)** | **0.592** |
| **PNI** |  |  |  |  |  |  |  |
| **Median (range)** | **41.9 (22.6-57.0)** | **39.1 (23.5-53.5)** | **0.154** |  | **40.9 (23.5-57.0)** | **40.1 (22.6-51.5)** | **0.431** |
| **NLR** |  |  |  |  |  |  |  |
| **Median (range)** | **4.5 (1.8-24.3)** | **4.3 (1.6-31.2)** | **0.924** |  | **4.2 (1.6-31.2)** | **4.7 (1.8-24.3)** | **0.413** |

| **Factor** | **Low-CYFRA** | **High-CYFRA** | **P-value** |  | **Low-LDH** | **High-LDH** | **P-value** |
| --- | --- | --- | --- | --- | --- | --- | --- |
|  | **n = 40** | **n = 58** |  |  | **n = 72** | **n = 28** |  |
| **Gender** |  |  |  |  |  |  |  |
| **Male / Female** | **33 / 7** | **47 / 11** | **1.000** |  | **56 / 16** | **25 / 3** | **0.260** |
| **Age** |  |  |  |  |  |  |  |
| **Median (range), years** | **70 (54–80)** | **72 (48-82)** | **0.299** |  | **71 (48–82)** | **69 (57-80)** | **0.815** |
| **Smoking status** |  |  |  |  |  |  |  |
| **Never / ever or current** | **4 / 36** | **5 / 53** | **1.000** |  | **9 / 63** | **0 / 28** | **0.058** |
| **ECOG-PS** |  |  |  |  |  |  |  |
| **0 / 1** | **27 / 13** | **32 / 26** | **0.294** |  | **48 / 24** | **13 / 15** | **0.072** |
| **Pathology** |  |  |  |  |  |  |  |
| **Adenocarcinoma / Squamous / others** | **34 / 4 / 2** | **30 / 21/ 7** | **0.002** |  | **49 / 18 / 5** | **17 / 7 / 4** | **0.475** |
| **PD-L1 expression** |  |  |  |  |  |  |  |
| **1%< / 1%–49% / ≥50%** | **16 / 11 / 10** | **23 / 13 / 19** | **0.687** |  | **24 / 22 / 20** | **15 / 3 / 10** | **0.064** |
| **Stage** |  |  |  |  |  |  |  |
| **Ⅲ, ⅣA / ⅣB** | **26 / 14** | **19 / 39** | **0.002** |  | **42 / 30** | **4 / 24** | **<0.001** |
| **The number of metastases** |  |  |  |  |  |  |  |
| **Median (range)** | **1 (0-10 or more)** | **3 (0-10 or more)** | **<0.001** |  | **1 (0-10 or more)** | **5 (0-10 or more)** | **<0.001** |
| **Brain metastases** |  |  |  |  |  |  |  |
| **Yes / No** | **7 / 33** | **15 / 43** | **0.461** |  | **12 / 60** | **11 / 17** | **0.032** |
| **Liver metastases** |  |  |  |  |  |  |  |
| **Yes / No** | **1 / 39** | **20 / 38** | **<0.001** |  | **10 / 62** | **12 / 16** | **0.003** |
| **CRP** |  |  |  |  |  |  |  |
| **Median (range)** | **0.85 (0.03-24.8)** | **2.6 (0.03-36.3)** | **0.022** |  | **1.4 (0.03-24.8)** | **3.3 (0.05-36.3)** | **0.058** |
| **LDH** |  |  |  |  |  |  |  |
| **Median (range)** | **193 (141-453)** | **240 (124-1855)** | **0.001** |  | **-** | **-** |  |
| **CEA** |  |  |  |  |  |  |  |
| **Median (range)** | **5.0 (0.9-3314)** | **8.1 (1.0-2866)** | **0.142** |  | **6.6 (0.9-1424)** | **12.4 (1.0-3314)** | **0.324** |
| **CYFRA** |  |  |  |  |  |  |  |
| **Median (range)** | **-** | **-** |  |  | **3.2 (1.0-43.0)** | **10.8 (1.7-75.0)** | **<0.001** |
| **GNRI** |  |  |  |  |  |  |  |
| **Median (range)** | **96.0 (64.2-117.0)** | **90.4 (52.0-114.8)** | **0.026** |  | **94.3 (52.0-117.0)** | **88.0 (67.0-114.8)** | **0.068** |
| **PNI** |  |  |  |  |  |  |  |
| **Median (range)** | **43.7 (27.7-57.0)** | **39.1 (22.6-53.5)** | **0.009** |  | **41.7 (23.5-57.0)** | **38.5 (22.6-53.5)** | **0.227** |
| **NLR** |  |  |  |  |  |  |  |
| **Median (range)** | **4.5 (1.6-23.6)** | **4.3 (1.6-31.2)** | **0.885** |  | **4.3 (1.6-31.2)** | **5.0 (1.6-18.3)** | **0.812** |

| **Factor** | **Number of metastases<3** | **Number of metastases>3** | **P-value** |  | **Low-NLR** | **High-NLR** | **P-value** |
| --- | --- | --- | --- | --- | --- | --- | --- |
|  | **n = 56** | **n = 44** |  |  | **n = 81** | **n = 19** |  |
| **Gender** |  |  |  |  |  |  |  |
| **Male / Female** | **45 / 11** | **34 / 5** | **0.297** |  | **65 / 16** | **16 / 3** | **1.000** |
| **Age** |  |  |  |  |  |  |  |
| **Median (range), years** | **70 (53–82)** | **72 (48-81)** | **0.911** |  | **72 (48–82)** | **70 (50-77)** | **0.160** |
| **Smoking status** |  |  |  |  |  |  |  |
| **Never / ever or current** | **8 / 48** | **1 / 43** | **0.073** |  | **8 / 73** | **1 / 18** | **1.000** |
| **ECOG-PS** |  |  |  |  |  |  |  |
| **0 / 1** | **38 / 18** | **23 / 21** | **0.149** |  | **50 / 31** | **11 / 8** | **0.798** |
| **Pathology** |  |  |  |  |  |  |  |
| **Adenocarcinoma / Squamous / others** | **37 / 15 / 4** | **29 / 10 / 5** | **0.738** |  | **52 / 22 / 7** | **14 / 3 / 2** | **0.607** |
| **PD-L1 expression** |  |  |  |  |  |  |  |
| **1%< / 1%–49% / ≥50%** | **19 / 16 / 17** | **20 / 9 / 13** | **0.489** |  | **32 / 19 / 24** | **7 / 6 / 6** | **0.900** |
| **Stage** |  |  |  |  |  |  |  |
| **Ⅲ, ⅣA / ⅣB** | **46 / 10** | **0 / 44** | **<0.001** |  | **38 / 43** | **8 / 11** | **0.801** |
| **The number of metastases** |  |  |  |  |  |  |  |
| **Median (range)** | **-** | **-** |  |  | **2 (0-10 or more)** | **2 (0-10 or more)** | **<0.001** |
| **Brain metastases** |  |  |  |  |  |  |  |
| **Yes / No** | **6 / 50** | **17 / 27** | **0.002** |  | **19 / 62** | **4 / 15** | **1.000** |
| **Liver metastases** |  |  |  |  |  |  |  |
| **Yes / No** | **3 / 53** | **19 / 25** | **<0.001** |  | **14 / 67** | **8 / 11** | **0.030** |
| **CRP** |  |  |  |  |  |  |  |
| **Median (range)** | **1.4 (0.04-18.8)** | **3.3 (0.03-36.3)** | **0.094** |  | **1.4 (0.03-18.7)** | **6.5 (0.26-36.3)** | **<0.001** |
| **LDH** |  |  |  |  |  |  |  |
| **Median (range)** | **195 (124-453)** | **276 (138-1855)** | **0.001** |  | **206 (124-1819)** | **241 (177-1855)** | **0.050** |
| **CEA** |  |  |  |  |  |  |  |
| **Median (range)** | **4.5 (0.9-493.2)** | **11.3 (1.0-3314)** | **0.015** |  | **5.8 (0.9-3314)** | **11.0 (1.4-1225)** | **0.311** |
| **CYFRA** |  |  |  |  |  |  |  |
| **Median (range)** | **2.6 (1.0-34.4)** | **8.0 (1.0-75.0)** | **<0.001** |  | **5.3 (1.0-74.0)** | **5.3 (1.0-75.0)** | **0.898** |
| **GNRI** |  |  |  |  |  |  |  |
| **Median (range)** | **94.5 (52.0-116.7)** | **90.2 (65.6-117.0)** | **0.209** |  | **93.6 (70.6-117.0)** | **82.3 (52.0-103.7)** | **0.001** |
| **PNI** |  |  |  |  |  |  |  |
| **Median (range)** | **41.2 (23.5-57.0)** | **39.9 (22.6-53.2)** | **0.453** |  | **41.9 (25.9-57.0)** | **32.0 (22.6-47.3)** | **<0.001** |
| **NLR** |  |  |  |  |  |  |  |
| **Median (range)** | **4.5 (1.6-31.2)** | **4.3 (1.6-24.3)** | **0.649** |  | **-** | **-** |  |

ECOG-PS: Eastern Cooperative Oncology Group performance status, PD-L1: programmed cell death 1 - ligand 1, NLR; neutrophil-to-lymphocyte ratio, GNRI; geriatric nutritional risk index PNI: prognostic nutritional index.
